# Effect of the stellate ganglion block on symptoms of ulcerative colitis: A case report

**DOI:** 10.1097/MD.0000000000026684

**Published:** 2021-07-23

**Authors:** Young Shin Kim, Jang Ho Song, Young Jun Kim, Kyung Joo Lee, Sun Hee Lee, Na Eun Kim

**Affiliations:** Department of Anesthesiology and Pain Medicine, Inha University Hospital, Inha University School of Medicine, Inhang-ro 27, Jung-gu, Incheon, Korea.

**Keywords:** autonomic nervous system, Stellate ganglion block, ulcerative colitis

## Abstract

**Rationale:**

Chronic ulcerative colitis is an autoimmune disease in which epithelial injury continuously occurs in the colonic mucosa. While mesalazine (5-aminosalicylic acid) is used to treat ulcerative colitis, it can also cause liver failure, headaches, and abdominal pain; therefore, an alternative treatment is required. The purpose of this study was to evaluate the effectiveness of 80 stellate ganglion blocks in reducing pain and other symptoms in a patient with chronic ulcerative colitis.

**Patient concerns:**

A 54-year-old female patient with a history of ulcerative colitis was concerned with worsening symptoms, such as abdominal discomfort and bloody-mucous stools, over the past 3 years.

**Diagnoses:**

Oozing mucosal bleeding and a small amount of exudate were observed on colonoscopy; a diagnosis of ulcerative colitis was made upon histologic examination.

**Interventions and outcomes:**

A total of 80 stellate ganglion blocks were administered, after which the patient's symptom and pain level was decreased from 6 to 4 points on the numeric rating scale (11-point, 0 = no pain, 10 = worst pain imaginable). Improved clinical signs were observed on colonoscopy at a follow-up assessment.

**Lessons:**

The stellate ganglion block may be effective for the reduction of pain and other symptoms in patients with chronic ulcerative colitis.

## 1 Introduction

Ulcerative colitis (UC) is a disease that causes epithelial injury due to chronic mucosal inflammation in the colon and rectum.^[1]^ It has a prevalence rate of 505 per 100,000 people in the United States. While mesalazine (5-aminosalicyclic acid) is commonly used as a treatment, continuous administration may cause side effects such as headache, abdominal pain, and liver failure. Therefore, there is a need for the development of alternative treatments for ulcerative colitis.^[2]^

The relationship between the immune system and sympathetic nervous system is complex. For instance, it has been established that the sympathetic nervous system is involved in various aspects of immune system regulation, such as the control of differential T-helper (Th) 1 and Th2 cell responses, as well as the regulation of antigen-presenting cell movement.^[3]^ Autoimmune diseases, such as rheumatoid arthritis, multiple sclerosis, and Crohn's disease, are known to be associated with the hyperactivation of the sympathetic nervous system. A previous randomized controlled study conducted among patients with ulcerative colitis reported a 30-fold decrease in interleukin (IL)-8 levels with stellate ganglion block administration, compared to a control.^[4]^

The stellate ganglion block is the commonly performed treatment in pain clinics. The stellate ganglion is a star-shaped structure, which is fused to the inferior cervical sympathetic ganglion and first thoracic sympathetic ganglion; the latter is an efferent sympathetic nerve that supplies the head and upper neck.^[5]^

Herein, we report improvements in clinical signs and symptoms with the administration of 80 stellate ganglion blocks in a patient with chronic ulcerative colitis.

The patient has provided and agreed informed consent for publication of this report.

## 2 Case presentation

A 54-year-old female diagnosed with ulcerative colitis was admitted to the pain clinic, as she was concerned with worsening symptoms, such as abdominal discomfort and bloody-mucous stools, within the past 3 years. She had been previously admitted 20 years ago due to abdominal discomfort and mucous stools. An examination at that time revealed vascular pattern loss, mucosal thickening, and touch bleeding up to 10 cm above the anal margin; the patient was diagnosed with ulcerative colitis via tissue biopsy, and the symptoms were relieved with medications such as mesalazine.

**Figure 1:**
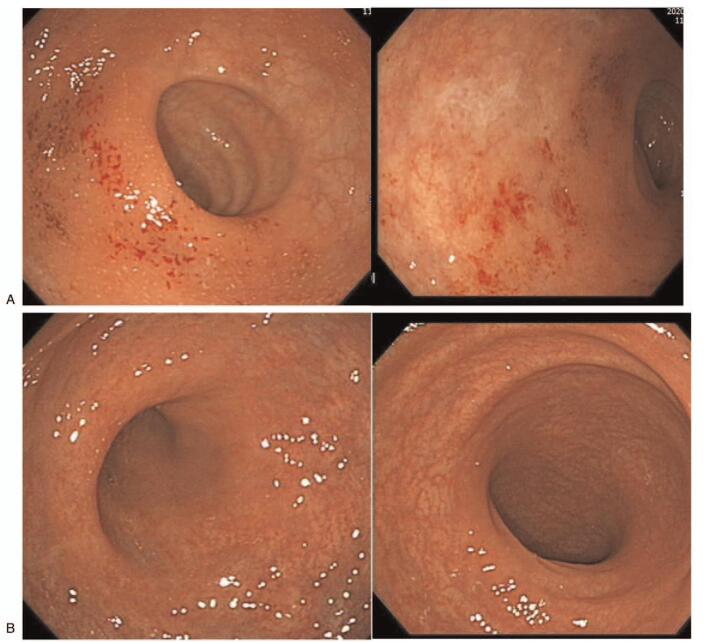
Endoscopic imaging findings. (A) Endoscopic imaging findings of the patient before the stellate ganglion block. Mucosal oozing. bleeding and tiny exudate. And moderate erytherma, abscent vascular pattern and erosions. (B) Endoscopic imaging findings of patient after 80 stellate ganglion blocks. Mucosal oozing bleeding and tiny exudate improved. And Intact vascular pattern.

At the current hospital admission, worsening inflammation was observed on colonoscopy. It was shown as the grade 2 on the Mayo endoscopic subscore with mucosal oozing and erosions on the colonoscopy. Laboratory findings included the following: fecal calprotectin quantitative test (<11.5 mg/kg), hemoglobin level (14.2 g/dL), leukocyte count (5,800/μL), platelet count (280,000/μL), erythrocyte sedimentation rate (7.0 mm/hour), C-reactive protein level (0.05 mg/dL), and antineutrophil cytoplasmic antibody test (negative findings). A stellate ganglion block was administered at both sides at intervals of an hour when visiting the hospital for 40 weeks. The stellate ganglion block was performed blindly using the anterior paratracheal technique, and the success of the block was confirmed by the increase in skin temperature of the index finger and the appearance of Horner's syndrome. Blood pressure, pulse rate, and oxygen saturation were checked during the procedure, and there was no case of hypotension or dyspnea after the procedure. The patient visited the hospital a total of 40 times, the stellate ganglion was blocked 40 times on the left side, 40 times on the right side, and a total of 80 times on the stellate ganglion block. Abdominal bloating, mucous stools, and posterior sensation were alleviated after the administration of total 80 blocks, the degree of abdominal pain decreased from 6 to 4 on a numerical rating scale (11-point, 0 = no pain, 10 = worst pain imaginable). Improved clinical signs with a normal vascular pattern were observed on colonoscopy at a follow-up assessment (Fig. 1). In laboratory findings, only the level of C-reactive protein was slightly reduced to 0.03 mg/dL, and there were no other significant changes. For 6 months after stellate ganglion blocks, the patient's symptoms and pain levels did not worsen.

## 3 Discussion

Ulcerative colitis is characterized by chronic and recurrent mucosal inflammation in the colon.^[1]^ It is most prevalent in the 30- to 40-year-old age group. Ulcerative colitis has been shown to be related to human leukocyte antigens such as hepatocyte nuclear factor 4 alpha and cadherin-1, and cigarette smoking is known to be a risk factor.^[1]^ The chronic inflammatory response in ulcerative colitis damages the mucosal barrier and is induced by the presence of luminal microflora.^[1]^ It occurs continuously through Th9 cells and IL-13, and innate and adaptive cellular immunity are known as pathogens.^[6]^ Sulfasalazine and steroids, which are used as treatments for ulcerative colitis, have been associated with both renal and hepatic injury; thus, there is a need for safer treatment options.^[2]^

A recent study reported reductions in pus, tenesmus, and IL-8 levels after the provision of 30 stellate ganglion blocks in patients with chronic ulcerative colitis.^[4]^ When stress or inflammation occurs, the autonomic nervous system shifts from a state of homeostasis to sympathetic dominance, thereby activating the immune system. As autoimmune diseases are related to the hyperactivation of the sympathetic nervous system, they may be treated with a stellate ganglion block, which reduces sympathetic nerve activation.^[7]^

The stellate ganglion block is the commonly performed treatment in pain clinics and has a low complication rate (1.7/1,000 procedures).^[5]^ The anterior paratracheal technique is the oldest and most commonly used approach for administering the stellate ganglion block. It involves the injection of local anesthetic at the anterior tubercle of C6, where the central cervical sympathetic ganglion is located. Recently, the use of an ultrasound guide for the stellate ganglion block has been described, in which the longus coli muscle is injected; as the procedure is monitored with real-time imaging, the risk of blood vessel injection and nerve damage is minimized. As the cervical sympathetic system has an anatomically different innervation and distribution on the left and right sides, varying effects are observed depending on the side on which the sympathetic nerve block is administered.^[5]^ While the right stellate ganglion block does not have a significant effect on the cardiovascular system, the left stellate ganglion block can cause regional wall motion abnormalities by reducing the contractile force of the myocardium. In addition, the side of block administration may also affect the QT interval: while QT prolongation may occur with a right block, a decrease in the QT interval may occur with a left block.^[8]^ The stellate ganglion blockade is known to affect not only the sympathetic nerve block, but also the immune and endocrine systems. However, the evidence remains controversial and studies are still underway. The stellate ganglion block has various effects, which include the following: improvement of cellular and immune function; regulation of an abnormally altered neuroendocrine system; restoration of sympathetic-vagus nerve imbalance due to increased sympathetic activity; and relief of excessive systemic nervous system activity.^[3]^ The stellate ganglion block can also be used for the treatment of ventricular electrical storms, as well as an alternative treatment for various diseases other than drugs. A previous study reported on the effectiveness of repeated stellate ganglion blocks in the resolution of seborrheic dermatitis.^[9]^ It can be considered that seborrheic dermatitis, which is caused by increased sebaceous gland secretion due to changes in host immune function, influenced autonomic nervous system homeostasis through stellate ganglion block. Another study reported a decrease in edema after repeated stellate ganglion blocks in patients with intractable lymphedema after breast cancer surgery. The reduction in edema was attributed to a decrease in post-capillary resistance, due to the effects of the stellate ganglion block on the immune system, as well as an increase in local body temperature.^[10]^ Furthermore, there is a current lack of evidence for the optimal dosage and frequency of administration for therapeutic purposes

Thus, based on prior research, we administered blocks on both sides at 1-hour intervals to achieve a complete sympathetic block; symptom relief was confirmed after more than 80 trials. After 80 stellate ganglion block procedures, symptoms of mucous stools and posterior sensation were relieved, abdominal pain decreased from 6 to 4 on a numerical rating scale, and changes in the colonoscopic image were confirmed.

Another study conducted among 50 patients with traumatic brain injury reported a significantly greater reduction in IL-6, IL-1β, tumor necrosis factor-α, and calcitonin gene-related peptide levels in the group receiving a stellate ganglion block, compared to a control group.^[11]^ A decrease in tumor necrosis factor-α (an inflammatory cytokine) levels was observed 2 days after the ganglion block procedure, and this was maintained for 7 days. In addition, p65 nuclear transcription factor-KB (an inflammatory factor) levels decreased 4 days after the procedure.^[11]^ A study utilizing an acute lung injury model in rabbits reported a decrease in the nociceptive response and excess sympathetic nervous system activity after stellate ganglion block administration.^[12]^ Reductions in plasma IL-6 and IL-10 levels were also observed. Another study evaluating the use of the stellate ganglion block in rats reported an immediate improvement in cochlear blood flow.^[13]^ Therefore, current evidence indicates that the dose and frequency of stellate ganglion blocks should be modified based on the cause and mechanism of the underlying disease. This warrants further investigation by future studies.

**Figure 2:**
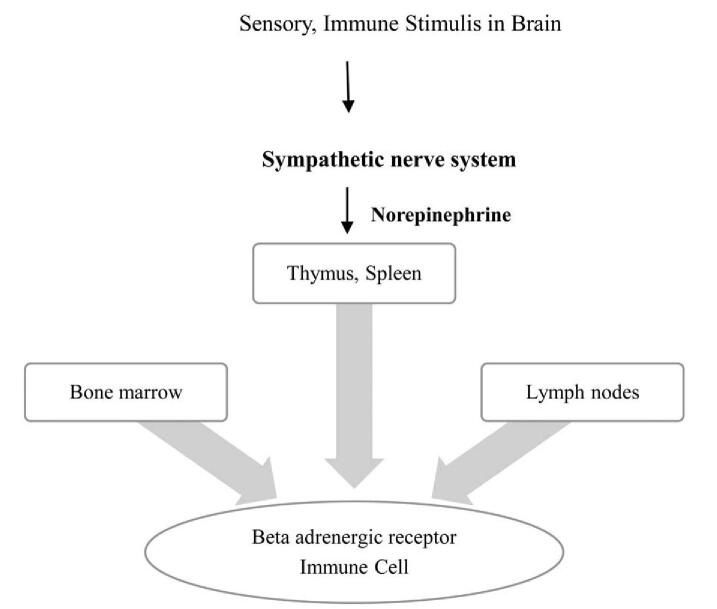
The sympathetic nerve system modulates the immune system. All immune organs receive sympathetic innervation from the sympathetic nerve. Sensory, immune stimulation is transmitted to the brain, and the sympathetic nervous system undergoes neuronal regulation in immune function. The sympathetic nerve suppresses the immune system, and norepinephrine regulates celluar activity.

The sympathetic nervous system regulates both the central nervous system and the immune system (Fig. 2). Prior studies have reported an increased secretion of catecholamines due to a hyperactive sympathetic nervous system, and their effects on the regulation of immune function in the primary lymphoid organs (bone marrow, thymus) and mucosal lymphoid tissues.^[14]^ The stimulation of beta-adrenergic receptors in Th1 cells can inhibit the production of Th1-type cytokines, which can also be indirectly inhibited by cytokines secreted by Th2 cells. Changes in sympathetic nervous system activity can lead to changes in noradrenergic innervation and persistent inflammation in immune organs, thus increasing the risk of immune-mediated diseases.^[14]^

Advantages of the stellate ganglion block include its easy anatomical accessibility and low incidence of side effects. The cervical sympathetic ganglia are divided into superior, middle, and stellate ganglion blocks. Among them, the superior sympathetic ganglia make the largest contribution to cerebral sympathetic activity and directly affect cerebral perfusion flow.^[15,16]^ In addition, it is known that the superior cervical sympathetic nerve has the greatest effect on cerebral vessel perfusion and the intrinsic central adrenergic pathway for the protection of the brain. However, in the case of the cervical sympathetic nerve, the influence of the brain and organs other than the stellate ganglion block is not well known because of their anatomical location and difficulty of access.

Additional studies are required to evaluate the effects of the cervical sympathetic nerve and the effect of the direct innervation of the celiac plexus block and the inferior mesenteric plexus block are also needed. When these studies are based, sympathetic-immune interactions become more apparent.

In this case, stellate ganglion block was performed 80 times while following the patient's symptoms, and when symptoms were relieved, a significant difference was also reported on the colonoscopy compared to before the procedure. The effect of the sympathetic nervous system and the immune system has not yet been clearly elucidated, and among patients with autoimmune diseases, the environment of chronic inflammatory changes due to changes in sympathetic nervous system activity in the immune organs is considered to be the cause. In this regard, stellate ganglion block is considered to be a good treatment method for autonomic homeostasis.

There has been a recent and rapid increase in the number of autoimmune diseases. While a wide range of studies have investigated the relationship between the sympathetic nervous system and immune system, the optimal drugs, doses, and treatment periods required for the stellate ganglion block and related procedures have not yet been established. Additional studies with larger sample sizes and a randomized controlled design are required.

## Author contributions

**Conceptualization:** Na Eun Kim.

**Data curation:** Young Shin Kim, Na Eun Kim, Jang Ho Song, Kyung Joo Lee.

**Funding acquisition:** Jang Ho Song.

**Methodology:** Kyung Joo Lee, Young Jun Kim.

**Project administration:** Young Jun Kim.

**Resources:** Sun Hee Lee.

**Software:** Jang Ho Song, Young Jun Kim.

**Validation:** Jang Ho Song.

**Writing – original draft:** Young Shin Kim, Na Eun Kim, Kyung Joo Lee.

**Writing – review & editing:** Na Eun Kim.
